# Evaluating a Project Extension for Community Health Outcomes Pediatric Behavioral Health Series in a Rural and Frontier State: An Exploratory Investigation

**DOI:** 10.1089/tmr.2022.0033

**Published:** 2023-03-17

**Authors:** Madeline P. Casanova, Ashley J. Reeves, Jonathan D. Moore, Seungho Ryu, Kathleen Palmer, Lachelle H. Smith, Jeffrey G. Seegmiller, Russell T. Baker

**Affiliations:** ^1^ University of Idaho WWAMI Medical Education, Idaho Office of Rural and Underserved Medical Research, Moscow, Idaho, USA.; ^2^ University of Idaho WWAMI Medical Education, Moscow, Idaho, USA.

**Keywords:** behavioral health, pediatrics, tele-mentoring, tele-education, continuing education

## Abstract

**Background:**

Idaho, a predominately rural state, has a high prevalence of mental illness with minimal access to care. Barriers in diagnosis and treatment of pediatric behavioral health disorders could be mitigated with an accessible and effective specialty training program.

**Methods:**

A 10-session Project Extension for Community Health Outcomes (ECHO) series was designed to expand provider knowledge about pediatric behavioral health conditions and improve perceived clinical practice skills. Pre- and postseries evaluation surveys and individual session evaluations were used to assess the program.

**Results:**

A total of 148 individuals attended at least 1 of the 10 sessions. Participants reported high satisfaction with individual sessions and indicated that attendance positively impacted their knowledge and competency. Participants also reported that the knowledge and skills gained from the series would benefit more than half of their patients or clients.

**Conclusion:**

The short ECHO series appears to be a viable and valuable option to provide Idaho providers with effective specialty training that is well attended and well received.

## Introduction

The prevalence of pediatric behavioral health disorders has been steadily increasing nationwide: a 24% increase in mental health-related emergency department visits for children ages 5–11 and a 31% increase in visits for children ages 12–17 years was reported between 2019 and 2020.^1^ The COVID-19 pandemic exacerbated current trends, as researchers have identified increased frequency of pediatric distress visits throughout the pandemic.^2–4^ Mental and behavioral health is of particular concern in rural areas where health care resources are scarce.^4^ Idaho, a predominately rural state, ranks 50th in the nation for youth health care resources and prevalence, indicating a high prevalence of youth mental illness and lower access to care.^5^

Unfortunately, only a fraction of youth diagnosed with a behavioral health disorder receive treatment.^5^ Researchers have identified numerous barriers to treatment: cost for care,^6^ access,^7^ cultural differences,^8^ and stigma surrounding mental health disorders.^9^ Barriers can be exacerbated in rural areas where there is a shortage of pediatricians^10^ and a significant portion of individuals living below the poverty line.^11^

In Idaho, the lack of access to providers (e.g., pediatrician, psychiatrist, primary care physician) for diagnosis and treatment is particularly concerning. As of 2015, residents in 66% of Idaho counties lacked access to a pediatrician, and residents in 70% of Idaho's counties lacked access to a psychiatrist, both with 0.0 per 10,000 children.^12^ Poor access to providers makes it difficult for youth residents to receive diagnoses for complex behavioral health concerns; challenges also persist after a diagnosis is received. For example, ∼58.1% of youth in Idaho who experienced a major depressive episode did not receive treatment.^13^ The cumulative situation results in many children suffering with behavioral health disorders who are unable to receive needed care.^13^

Another major barrier identified for rural areas is the lack of knowledge and competence among the available providers (e.g., primary care providers [PCPs]) to treat pediatric behavioral health concerns.^12^ Many PCPs lack access to specialty training and have not previously engaged with educational materials aimed at establishing competency with identifying and treating behavioral health disorders.^14^ Barriers and challenges in the diagnosis and treatment of pediatric behavioral health disorders could be mitigated by developing an accessible and effective specialty training program for health care professionals and care teams. Upscaling current provider knowledge and competency for diagnosing pediatric behavioral health disorders could serve as a mechanism to expeditiously reduce patient and provider barriers, decrease strain on health care system resources, and improve patient outcomes. One such option is Project Extension for Community Health Outcomes (Project ECHO).^15^

Project ECHO was designed to connect rural health care providers to subject matter experts to improve provider knowledge and skills to empower them to adequately care for patients in their communities with various complex health conditions.^15^ Programs are established and offered through online sessions with subject matter experts to attract a variety of health care providers across the state.

ECHO Idaho, part of the WWAMI (Washington, Wyoming, Alaska, Montana, Idaho) Medical Education Program at the University of Idaho, is the Project ECHO replication partner for the state of Idaho. ECHO Idaho was launched in 2018 and currently offers ongoing programming related to infectious disease, substance use disorders, and behavioral health.^16^ To meet the urgent and growing needs of the state, ECHO Idaho developed programming for pediatric behavioral health. The 10-session short series on pediatric behavioral health was designed with the goal of providing a training program that Idaho providers perceived as beneficial for upscaling their knowledge, confidence, and clinical skill to improve the diagnosis and treatment of pediatric behavioral health conditions.

While previous researchers have found that ECHO Idaho programs enhance knowledge, improve perceived patient care, develop a peer network across the state, and reduce barriers to attending advanced training and attaining continuing education credits,^17,18^ very little is known about the effects on provider knowledge and competence from participating in a 10-session Pediatric Behavioral Health short series, especially in a rural state such as Idaho. Therefore, the purpose of this article was to evaluate a 10-session Project ECHO Pediatric Behavioral Health (Peds BH) short series in Idaho, a predominately rural and frontier state.

## Methods

### ECHO Idaho Pediatric Behavioral Health Training

A pediatric behavioral health series was developed by ECHO Idaho and led by a panel of pediatric behavioral health experts in Idaho, including a psychologist, pediatrician, pharmacist, school nurse, pediatric psychiatrist, and parent advocate. The series was offered free of charge to health care providers who desired to expand their knowledge about pediatric behavioral health conditions and application to practice, and was marketed through the Idaho WWAMI and ECHO Idaho webpages, email messages to prior ECHO Idaho program attendees, and word of mouth.

The target audience recruited to participate in the series was Idaho PCPs (e.g., MD, PA, NP), but all other clinicians and behavioral health specialists were able to attend. Series advertisement included the offering of free continuing medical education credits to physicians, pharmacists, nurses, physician assistants, psychologist, counselors, marriage and family therapists, social workers, and alcohol and drug counselors (CADC/ACADC/CPS). The 1-h-long sessions were offered twice monthly from May 2021 to September 2021 and included a 30-min didactic lecture, led by a member of the expert panel, and a 30-min case-based discussion.

During the discussion, a deidentified patient case was presented by a health care provider attendee and the expert panel and audience members provided input regarding patient management. Following the session, the panel of experts also offered continued mentorship to the case presenter and provided treatment and medical care recommendations.

### Series evaluation

Pre- and postseries electronic surveys (Qualtrics, LLC, Provo, UT) were developed to assess perceived knowledge and competence related to pediatric behavioral health before and after attending the Project ECHO Idaho Pediatric Behavioral Health series. The survey items were grouped into three sections: (1) respondent demographics, (2) clinical practice application, and (3) perceived clinical practice benefits (i.e., knowledge and competence related to pediatric behavioral health conditions). In addition, for the preseries evaluation survey only, respondents were asked to indicate learning goal objectives. Survey item response types included: single answer, multiple answer, 5-point Likert scales (1 = not at all competent, 5 = extremely competent; 1 = not at all knowledgeable, 5 = extremely knowledgeable) and open-ended text answers.

The preseries survey was distributed through email before the launch of the series on May 3, 2021; data collection closed on May 13, 2021. The postseries survey was distributed after the completion of the series on September 23, 2021, and data collection closed on October 8, 2021.

### Single session evaluation

Individual sessions were also evaluated using an electronic survey (Qualtrics, LLC). The 9-item survey assessed satisfaction with a specific session as well as the impact of the individual sessions on clinical practice. Survey item response types included: single answer, sliding scale percentages, 5-point Likert scales (1 = strongly disagree, 5 = strongly agree), and open-ended text answers. Individuals who completed this survey were eligible to claim Continuing Education credits for each live session attended.

### Procedures

The project was certified exempt (i.e., approved) by the University Institutional Review Board. Health care providers completed a registration form on the ECHO Idaho website to attend the ECHO Idaho Peds BH series. Demographic information was collected in the registration form, and iECHO, a web-based program database developed by Project ECHO, was utilized to house attendee information (e.g., credentials, job title, primary practice location).

Before the launch of the ECHO Idaho Peds BH series, a link to the electronic preseries evaluation survey was sent to all providers who registered for the series. Following the completion of each individual series session, attendees were sent a link to complete the single session evaluation survey. Following the completion of the ECHO Idaho Pediatric Behavioral Health series, all individuals who had attended at least one individual session were invited to participate in the postseries evaluation survey. Individuals who completed the pre- and postsurveys were sent ECHO Idaho branded items (i.e., water bottle, journal) as an incentive.

### Data analysis

Data from the series evaluation and single-session evaluation surveys were exported from Qualtrics for analysis. The Statistical Package for the Social Sciences version 25 (SPSS, Inc., IBM Corp., Armonk, NY) was used to compute descriptive statistics. Duplicate entries were removed; however, incomplete cases (e.g., items that were skipped due to lack of applicability to respondent profession) were retained.

Descriptive data were reported as mean (*M*) ± standard deviation (SD) for continuous variables, or as a percentage for categorical variables. Means and percentages were calculated based on the valid *N* of each respective item. Likert scale responses were classified as continuous variables for analysis. A general inductive approach was used for all qualitative data analysis; two members of the team independently analyzed the data and a third team member consulted in the event a discrepancy occured.^19^ Responses were analyzed individually from the preseries evaluation survey, individual session evaluation survey, and the postseries evaluation survey. Additionally, we did a preliminary comparative analysis of data from individuals who responded to both the preseries and postseries evaluation survey.

## Results

### Series evaluation

#### Attendee demographic information

A total of 148 unique attendees participated across the 10 ECHO Idaho Pediatric Behavioral Health sessions. Full demographic information is presented in Table 1. A total of 108 (72.9%) attendees were practicing health care providers across a variety of specialty areas, with the most common being social work (*n* = 28, 18.9%). Most attendees were from Idaho (*n* = 130; 87.8%); however, 18 (12.2%) attendees were from out of state (e.g., Alaska, Colorado, Washington). Idaho attendees represented 21 different municipalities and 81 different health care organizations and had practiced for an average of 15.6 years (SD = 8.62). Additionally, all seven Idaho public health districts (PHDs) were represented. Series sessions averaged 36 attendees (range = 23–50 participants) per session; individual session attendance is provided in Table 2.

**Table 1.** Demographic Information

Variable	Series attendance, ***N*** (%)	Preseries respondent, ***N*** (%)	Postseries respondent, ***N*** (%)
Sex			
Male	11 (7.4)	3 (18.8)	5 (15.6)
Female	83 (56.1)	13 (81.3)	27 (84.4)
Prefer not to answer	3 (2.0)	—	—
Unknown	51 (34.5)	—	
Race/ethnicity			
White	87 (58.4)	14 (87.5)	30 (90.9)
Black or African American	3 (2.0)	2 (12.5)	—
Asian	—	—	1 (3.0)
Hispanic or Latino or Spanish Origin	4 (2.7)	—	2 (6.1)
Other	1 (0.07)	1 (6.3)	—
Prefer not to answer	3 (2.0)	—	—
Unknown	51 (24.2)	—	—
Public health district			
District 1	13 (10.0%)	2 (12.5)	6 (10.9)
District 2	12 (9.2%)	4 (25.0)	6 (10.9)
District 3	8 (6.2%)	1 (6.3)	13 (23.6)
District 4	83 (63.6%)	4 (25.0)	16 (29.1)
District 5	4 (31%)	6 (37.5)	2 (3.6)
District 6	3 (2.3%)	—	3 (5.5)
District 7	7 (5.4%)	3 (18.8)	9 (16.4)
Primary professional credential			
Physician (MD/DO)	17 (11.5%)	2 (12.5)	3 (9.4)
Physician assistant	7 (4.7%)	1 (6.3)	2 (6.3)
Nurse practitioner	8 (5.4%)	2 (12.5)	5 (15.6)
Nurse	24 (21.6%)	2 (12.5)	5 (15.6)
Pharmacist	3 (2.0%)	1 (6.3)	1 (3.1)
Psychologist	2 (1.4%)	1 (6.3)	—
Social worker	28 (18.9%)	3 (18.8)	12 (37.5)
Counselor	14 (9.5%)	1 (6.3)	1 (3.1)
Health profession student	10 (6.8%)	1 (6.3)	1 (3.1)
Other	35 (23.6%)	2 (12.5)	2 (6.3)

Participant demographic information for series attendance and survey respondents.

**Table 2.** Series Attendance

Title of session	No. of participants
Suicide Assessment and Medication Management	47
Screening Tools and Comorbid Conditions	34
Management of Anxiety and Depression	50
ADHD	33
Adolescent Eating Disorders	39
Strengthening Primary Care and School Health Systems	36
Types of Therapy	23
Managing Crisis Care for Families with Children with Serious Emotional Disturbance	37
Health Care Transition	33
Health Care Transition from the Parent Perspective	29

ADHD, attention-deficit/hyperactive disorder; ECHO Idaho Pediatric Behavioral Health series attendance by session.

### Preseries evaluation survey

#### Respondent demographics

Sixteen individuals (10.8%, 16/148) responded to the preseries survey, representing 13 counties and six PHDs (Table 1). Respondents were 48.5 ± 13.2 years old (range = 25–65), with an average of 11 ± 10 years of clinical practice experience (range = 0–35 years); the majority were female (*n* = 13, 81.3%) and White (*n* = 14, 87.5%). A variety of professions, employment settings, and clinical practice populations were represented (Tables 1 and 3).

**Table 3.** Series Evaluation Demographic Information

	Preseries, ***N*** (%)	Postseries, ***N*** (%)
Practice setting(s)		
Office-based Group Practice	5 (31.3)	7 (21.9)
Office-based Solo Practice	—	2 (6.3)
Specialty Substance Use Disorder Treatment Facility	1 (6.3)	1 (3.1)
Hospital or Health System	4 (25.0)	3 (9.4)
Federally Qualified Health Center	1 (6.3)	1 (3.1)
Rural Health Clinic	1 (6.3)	1 (3.1)
Community Health Agency	3 (18.8)	3 (9.4)
Academic Medical Center	1 (6.3)	1 (3.1)
School	—	6 (18.8)
Other	5 (31.3)	7 (21.9)
Practice population(s)		
People with Mental Health Problems	13 (81.3%)	28 (87.5%)
Low-Income Residents	12 (75.0%)	26 (81.3%)
People with Substance Use Disorders	11 (68.8%)	22 (68.8%)
Rural Residents	10 (62.5%)	21 (65.6%)
Hispanics/Latinos	8 (50.0%)	17 (53.1%)
American Indian/Tribal Communities	4 (25.0%)	11 (34.4%)
Refugees/Resettlement Residents	2 (12.5%)	9 (28.1%)
Other	3 (18.8%)	1 (4.0%)

Practice setting and population demographic information.

#### Clinical application

Respondents indicated that 35.0% of their time (range = 0–100%) was spent treating pediatric patients and 39.0% of their time (range = 0–100%) was spent treating pediatric patients with behavioral health concerns (range = 0–100%). They estimated that 57.6% (range = 0–100%) of their patients or clients were expected to benefit from the information provided in the sessions.

#### Perceived clinical practice benefits. 

##### Perceived knowledge

Respondents also rated their perceived knowledge, ranging from *not at all knowledgeable* (1) to *extremely knowledgeable* (5) in 11 practice areas. Results for all items are presented in Table 4. Generally, respondents reported moderate knowledge of pediatric anxiety (3.56 ± 0.73); low knowledge about prescribing medications for pediatric behavioral health problems (2.21 ± 1.42); and low to some knowledge regarding nonpharmacological treatment modalities (2.63 ± 1.26), adolescent eating disorders (2.81 ± 0.75), and pediatric behavioral health referral practices (2.88 ± 0.81).

**Table 4.** Clinical Practice Impact

Knowledge level	Preseries, ***M*** (SD)	Postseries, ***M*** (SD)	Percent change
Suicide Assessment	3.37 (1.15)	3.88 (0.87)	15.1%
Prescribing Medication	2.21 (1.42)	2.41 (1.47)	9.0%
Screening Tools	3.13 (0.89)	3.53 (0.72)	12.8%
Comorbid Conditions	3.06 (1.00)	3.39 (0.88)	10.8%
Anxiety	3.56 (0.73)	3.84 (0.74)	7.9%
Depression	3.44 (0.73)	3.91 (0.69)	13.7%
ADHD	3.25 (0.78)	3.77 (0.76)	16.0%
Adolescent Eating Disorders	2.81 (0.75)	3.13 (0.79)	11.4%
Serious Emotional Disturbances	2.94 (1.18)	3.10 (1.01)	5.4%
Nonpharmacological Treatment Modalities	2.63 (1.26)	2.87 (0.94)	9.1%
Referral Practices	2.88 (0.81)	3.53 (1.02)	22.6%

**Competency level**	**Pre-series**	**Post-series**	**Percent change**
**My Current Ability to…**	***M* (SD)**	***M* (SD)**	
Diagnose and differentiate between pediatric mental health conditions	3.19 (1.11)	3.38 (0.86)	6.0%
Use evidence-based screening tools for pediatric behavioral health	2.88 (1.15)	3.59 (0.71)	24.7%
Deliver evidence-based pediatric behavioral interventions	3.06 (1.06)	3.66 (0.90)	19.6%
Provide education and resources regarding pediatric behavioral health conditions	2.88 (0.89)	3.48 (1.03)	20.8%
Effectively prescribe psychotropic medications for pediatric behavioral health conditions	2.54 (1.39)	2.46 (1.50)	−3.1%
Persuade families to follow through with pediatric behavioral health referrals	3.19 (0.98)	3.71 (0.94)	16.3%

**To Manage…**	***M* (SD)**	***M* (SD)**	**Percent change**
Suicide	3.27 (1.10)	3.75 (0.98)	14.7%
Medications	2.15 (1.41)	2.77 (1.41)	28.8%
Comorbid Conditions	2.80 (1.21)	3.28 (0.89)	17.1%
Anxiety	3.53 (0.74)	3.78 (0.79)	7.1%
Depression	3.47 (0.64)	3.78 (0.79)	8.9%
ADHD	3.27 (0.80)	3.63 (0.94)	11.0%
Adolescent Eating Disorders	2.60 (0.91)	2.94 (0.91)	13.1%
Serious Emotional Disturbances	2.80 (1.21)	2.97 (1.03)	6.1%
Crisis Care for Families	2.53 (1.19)	3.09 (1.06)	22.1%
Nonpharmacological Treatment Modalities	2.60 (1.30)	3.00 (0.95)	15.4%

Individual results pre and postseries regarding the impact on perceived clinical practice.

SD, standard deviation.

##### Perceived competence

Respondents rated their competence, ranging from *not at all competent* (1) to *extremely competent* (5), in 6 areas of ability and 10 areas of condition management before attending the series. Generally, respondents indicated moderate levels of perceived competence in their abilities to diagnose and differentiate between pediatric behavioral health conditions (3.19 ± 1.11) and their ability to persuade families to follow through with pediatric behavioral health referrals (3.19 ± 0.98); low levels of competence with medications (2.15 ± 1.41) and their ability to effectively prescribe psychotropic medications (2.54 ± 1.39); and moderate competence managing anxiety concerns with pediatric patients (3.53 ± 0.74). Respondents also reported moderate competence in managing concerns with pediatric suicide (3.27 ± 1.10) and attention-deficit/hyperactive disorder (ADHD) (3.27 ± 0.80), with lower levels of competence in managing crisis care for families (2.53 ± 1.19). Detailed results are provided in Table 4.

#### Learning goal objectives

Respondents described learning goals for the series and five main themes were identified: (1) Enhance Knowledge; (2) Evidence-Based Practice; (3) Clinical Application; (4) Patient Populations; and (5) Peer Networking. The “Enhance Knowledge” theme encompassed participants' desire to expand knowledge in relevant areas related to their profession. One participant stated, “I currently practice in adult mental health, but would like to expand my knowledge to be a more well-rounded provider.” Participants also wished to learn about evidence-based practice (i.e., “Evidence-Based Practice”) to “stay on top of mainstream approaches to [patient] care” and to “[keep] up to date with the evidence base.” Participants also hoped to apply the learned information to their clinical practice (i.e., “Clinical Application”). Representative statements included gaining “tools to make more accurate diagnoses,” and learning “skills to be more informed and better prepared to help families in [their] practice.”

The ‘Peer Networking’ theme described the desire for collaboration between health care providers. Many participants wanted to connect with colleagues across the state; representative statements included, “being increasingly aware of other professionals working in the community in this area” and implementing “interdisciplinary treatment.”

### Single-session evaluation surveys

Evaluation surveys were collected for 9 of the 10 sessions. A total of 141 individual session evaluation surveys were completed across the nine sessions; an average of 16 surveys were completed per session (range = 11–26). All respondents were satisfied (100%) with the sessions, and most (85.2%) indicated the presented information was new and impactful for their professional/clinical practice. Respondents estimated the presented information would impact ∼50.2% of their patients, while also indicating the sessions positively impacted their perceived knowledge and clinical practice (Table 5).

**Table 5.** Single-Session Evaluation Survey

Statement	Overall, mean (SD)
Increased my overall knowledge.	4.59 (0.51)
Improved my understanding of an interdisciplinary approach to care.	4.54 (0.55)
Enhanced my ability to use best practices when caring for my patients/clients.	4.50 (0.59)
Provided a peer network for me to utilize in my patient/client care.	4.48 (0.67)
Presented information that enhances my patient/client care.	4.55 (0.57)
Provided information I will incorporate into my practice.	4.52 (0.59)

Mean responses for items on the individual session evaluation survey averaged across all session evaluations.

### Postseries evaluation survey

#### Respondent demographics

A total of 32 (21.6%, 32/148) Idaho health care professionals who attended at least 1 session responded to the postseries survey; respondents represented 25 counties, 19 of which are classified as rural, across 7 PHDs. A variety of professions, employment settings, and clinical practice populations were represented (Tables 1 and 3). Overall, respondents were 45.9 ± 12.0 years old (range = 28–66), with an average of 15.0 ± 11.7 years of clinical practice experience (range = 1–40). Respondents reported that ∼61.5% of their clinical time (range = 10–100%) is spent treating pediatric patients/clients and ∼40.7% of their time (range = 10–95%) is spent treating pediatric patients/clients with behavioral health concerns. Finally, of the postseries individuals that responded to the question, the majority (*n* = 17, 54.8%) indicated the session(s) provided new and relevant information with definite impact on professional/clinical practice.

#### Clinical application perceptions

Respondents (*n* = 30) reported that ∼51.8% (range = 9–100%) of their patients or clients would benefit from the session information, and that they would use this information with their patients or clients a couple of times per week. Of the 22 that responded, 90.9% (*n* = 20) indicated that they were more willing to treat pediatric patients with behavioral health concerns because of their participation. Additionally, 15 (68.2%) of the 22 providers reported they could accept new patients at the time, and of the 15, 93.3% (*n* = 14) indicated they were more willing to accept new pediatric patients because of their participation in the series. Furthermore, of the 28 individuals who responded, 96.4% (*n* = 27) reported they believed they will be more effective at treating pediatric patients with behavioral health concerns because of participation in the ECHO Idaho series. One respondent did not believe attending increased their effectiveness but rather reaffirmed their clinical practice.

#### Perceived clinical practice benefits

A total of 24 (75.0%, 24/32) respondents completed items related to the perceived clinical impact, impact on knowledge, and competency. Twenty-three out of 24 respondents (95.8%) indicated their knowledge, competency, and practice had changed because of their participation in this ECHO Idaho series.

##### Perceived knowledge

Respondent knowledge scores are provided in Table 4. Overall, all areas of practice were rated at 3 (i.e., somewhat competent) or greater, except prescribing medication (2.41 ± 1.47) and nonpharmacological treatment modalities (2.87 ± 0.94) following completion of the ECHO Idaho Peds BH series.

##### Perceived competence

Respondent competency scores are provided in Table 4. Overall, respondents indicated moderate-to-high levels of competence in all areas after completing the ECHO Idaho training, except those related to medication prescription (2.46 ± 1.50). Perceived competency was highest for anxiety (3.78 ± 0.79) and depression (3.78 ± 0.79) and lowest for medications (2.77 ± 1.41).

### Pre/post exploratory comparison

A total of seven individuals who completed both the pre- and postseries survey and had attended at least one Peds BH session were included in the pre/post comparison analysis. Perceived competence and knowledge levels were moderately to substantially improved across most of the assessed areas and conditions (Table 6). Perceived knowledge increased in all areas, except prescribing medication for behavioral health conditions. The most substantial changes were in respondent competence in managing crisis care for families (46.9% improvement) and providing education and resources for pediatric mental health conditions (44.4% improvement). Changes were also reported in respondents' perceived ability to use evidence-based screening tools (36.9% improvement) and knowledge of referral practices (29.7% improvement) for pediatric behavioral health conditions. There was a perceived decrease in knowledge and competence related to prescribing medications while ability to diagnose and differentiate between pediatric mental health conditions remained the same.

**Table 6.** Pre/Post Comparisons for Knowledge and Competence Items

Variable	Preseries, ***M*** (SD)	Postseries, ***M*** (SD)	Percent change
Knowledge Level			
Suicide Assessment	3.29 (0.95)	3.57 (0.98)	8.5%
Prescribing Medication	2.60 (1.52)	2.33 (1.75)	−10.4%
Screening Tools	3.00 (0.58)	3.29 (0.95)	9.7%
Comorbid Conditions	3.00 (0.82)	3.17 (1.33)	5.7%
Anxiety	3.43 (0.54)	3.71 (0.76)	8.2%
Depression	3.43 (0.54)	3.71 (0.76)	8.2%
ADHD	3.29 (0.49)	3.83 (0.98)	16.4%
Adolescent Eating Disorders	2.86 (0.38)	3.00 (0.82)	4.9%
Serious Emotional Disturbances	2.86 (0.90)	3.17 (1.47)	10.8%
Nonpharmacological Treatment Modalities	2.57 (0.98)	3.17 (1.33)	23.3%
Referral Practices	2.86 (0.69)	3.71 (1.25)	29.7%
Competence			
My Current Ability to…			
Diagnose and differentiate between pediatric mental health conditions	3.29 (0.76)	3.29 (1.11)	0.0%
Use evidence-based screening tools for pediatric behavioral health	2.71 (0.76)	3.71 (0.95)	36.9%
Provide education and resources regarding pediatric behavioral health conditions	2.57 (0.54)	3.71 (1.11)	44.4%
Deliver evidence-based pediatric behavioral interventions	3.00 (0.82)	3.43 (1.72)	14.3%
Effectively prescribe psychotropic medications for pediatric behavioral health conditions	2.40 (1.34)	2.14 (1.68)	−11.8%
Persuade families to follow-through with pediatric behavioral health referrals	3.29 (1.11)	3.57 (1.27)	8.5%
To Manage…			
Suicide	3.14 (0.90)	3.71 (1.11)	18.0%
Medications	2.40 (1.34)	2.57 (1.62)	7.1%
Comorbid Conditions	3.00 (0.82)	3.29 (0.76)	9.7%
Anxiety	3.29 (0.49)	3.71 (0.76)	12.8%
Depression	3.29 (0.49)	3.71 (0.76)	12.8%
ADHD	3.14 (0.69)	3.71 (0.95)	18.2%
Adolescent Eating Disorders	2.71 (0.49)	2.86 (1.07)	5.5%
Serious Emotional Disturbances	2.57 (0.79)	3.14 (1.35)	22.2%
Crisis Care for Families	2.43 (0.79)	3.57 (1.27)	46.9%
Nonpharmacological Treatment Modalities	2.57 (0.98)	3.00 (1.29)	16.7%

Exploratory comparison of respondent pre- and postsurvey competence and knowledge scores.

## Discussion

Pediatric behavioral health has become an increasing concern across the nation. The increased prevalence of pediatric behavioral health conditions is especially concerning in rural states such as Idaho, where access to care can be a challenge due to geographic constraints and the severe shortage of mental health providers. Furthermore, providers often do not feel competent in treating behavioral health conditions.^12,14^ Therefore, an intervention is needed to increase competence, knowledge, and peer networking among health care providers surrounding the treatment and management of pediatric behavioral health conditions to allow Idaho children to receive appropriate and effective care.

ECHO Idaho provides an opportunity for health care providers, particularly those in rural communities, to access the necessary specialty training, be a part of a supportive community of providers, and improve their knowledge and competence to enhance patient outcomes.^17,18^ The purpose of this study was to assess perceptions of series impact on knowledge and competence related to pediatric behavioral health, as well as general assessment of the ECHO Idaho Peds BH series.

### Preseries results

Survey respondents indicated a desire to build a peer network of providers across the state and to learn about a range of topics that would inform their patient/client care. The primary patient/client populations treated included individuals with mental health problems, low-income residents, individuals with substance use disorders, and rural residents. The respondents, however, reported a general lack of (i.e., low to moderate) competence and knowledge across a variety of clinical practice areas and behavioral health conditions, particularly with respect to medication prescription and management. Respondents felt most competent and knowledgeable with pediatric anxiety and depression.

Thus, the preseries findings further supported the need for pediatric behavioral health programming through ECHO Idaho for Idaho health care providers. Not only did providers not feel competent or knowledgeable in a wide variety of conditions and practice areas, but they also served patient populations that otherwise may not have access to a provider equipped to manage behavioral health conditions. With the responsibility of care falling on PCPs when specialists are not accessible, it is imperative that existing providers have the skills necessary to treat these patients. ECHO Idaho not only provides the opportunity to increase knowledge and competence in treating behavioral health conditions, but also allows providers to build a wider referral network to give more optimal care to patients in need of behavioral health services.

### Program participation and attendance

The ECHO Idaho Peds BH series was well attended and was able to reach providers across all seven Idaho PHDs. The ability to reach providers across Idaho is consistent with other ECHO Idaho series.^17,18^ The ECHO Idaho Peds BH series was also effective in attracting a diverse group of participants. Despite only being a 10-week series, 148 unique individuals from 81 different health organizations attended a session; in comparison, year-long ECHO Idaho series have produced similar participation turnouts (e.g., 178 unique participants from 101 health organizations).^17,18^

Our results are consistent with prior ECHO findings that these series are effective at recruiting participants across multiple health professions, which supports interdisciplinary care and more effective practice.^17,18^ However, our data also indicate a unique benefit of the ECHO Idaho Peds BH series: the series attracted health profession students who can now incorporate this information into their professional training, which allows them to implement it into clinical practice immediately upon joining the workforce.

The ECHO Idaho BH series analyzed, while shorter in number of sessions offered (i.e., 10 sessions), reached a large audience of Idaho health care stakeholders. Longer duration ECHO series (i.e., those with >10 sessions in a series) have been well attended on average (26 and 23 attendees per session in prior reports for behavioral health and opioid series, respectively);^17,18^ however, average session attendance at these other series was not as high as the average attendance for the ECHO Idaho Peds BH series (36 attendees per session).

Our results also indicate a high level of participation when compared with other Project ECHO efforts in other states or countries from larger populous areas on similar topic areas. For example, the ECHO Ontario Mental Health Program, which included site agreements with a minimum of 70% attendance from 26 sites, had 131 unique participants (over a 32-week period), with an average session attendance of 34.^20^ Similarly, over a 12-month period (24 sessions), 336 unique participants attended the Ontario Pediatric Palliative Care Project ECHO, with an average session attendance of 32.^21^

### Participant satisfaction

Overall, the ECHO Idaho series have an established track record of excellent participation (i.e., session attendance often meets or exceeds more populous states) with highly satisfied attendees.^17,18^ The ECHO Idaho Peds BH series was no different, as overall satisfaction (100%) with the sessions met or exceeded other Project ECHO series in Idaho^17,18^ or those in other states.^20,22^ Furthermore, 97% of postseries evaluation respondents indicated the information provided was new and relevant information for clinical practice.

The series was also able to support efficient participation (e.g., reduce barriers to attendance) due to delivery mode and a valued experience, while also providing opportunities to earn continuing education credits and establish peer networks. While access to continuing education programs and peer networks, particularly for rural and frontier providers, is often a challenge,^23^ our results indicate the ECHO Idaho program can be a viable and valuable option for providers in remote or underserved areas to receive specialty education and connect with others. ECHO Idaho participation connects health professionals in Idaho and creates a peer network that can be utilized in their patient/client care.^17,18^

An Idaho peer network is particularly important to improve care because of the geographic barriers of the state, which limit patient access to providers, and because peer networks can support provider retention and recruitment.^24^ Future efforts should focus on supporting the established peer network with further training and examining how the established network supports patient care.

### Perceived clinical practice benefits

After attending at least one session of the series, respondents reported positive feedback and indicated perceived improvement in most areas of knowledge, competence, and clinical practice application. Most respondents (96.4%) believed they were more effective at treating pediatric patients with behavioral health conditions because of their attendance in the series and estimated that the information would impact over 50% of their patients/clients. Our results are similar to other Project ECHO efforts that have reported increased knowledge and self-efficacy after attending series sessions.^20,22^

Of the postseries survey responses, the largest perceived knowledge area change was in referral practices, which one participant described as learning to “lean on referrals.” This result is similar to a Pediatric Behavioral Health Project ECHO that found participants in the program had significant increases regarding knowledge of local referral resources and how to support referral follow through.^22^ Participants who completed both the pre- and postseries surveys indicated their greatest growth knowledge area was related to referral practices, which supports the value of peer networking and interdisciplinary learning in ECHO Idaho to help optimize collaborative and interdisciplinary care.

It is also important to note that respondents reported perceived improvements in knowledge of (23%) and ability to manage (17%) nonpharmacological treatment modalities, which may enhance the ability of providers to care for their patients. The largest perceived competence increases also potentially support enhanced care as respondents cited improved ability to provide education and resources, use evidence-based screening tools, and manage crisis care for families.

Again, our results are consistent with previous literature that has identified Project ECHO series increase perceived knowledge and competence related to screening tools.^22,25^ The reported competency improvements in managing serious emotional disturbances and providing education and resources for pediatric behavioral health conditions are also consistent with other Project ECHO findings.^22^ Advanced training is important to support provider confidence, competence, and patient care improvement; confident providers are more likely to perform new clinical tasks, demonstrate resiliency when challenges arise, and improve performance.^25–27^

In contrast to the overall postseries survey results, knowledge about medication prescription and the ability to effectively prescribe medications did not improve. The results are not consistent with previous literature;^22,25^ however, the findings could be explained by several factors. One possible explanation is that most respondents answered questions about medication management and prescription, regardless of whether they were able to prescribe medications. Therefore, much of the information may have been new or irrelevant to those providers, which could result in scores not changing or decreasing after being exposed to new information.

Another possible explanation is the lack of exposure to prescription medication content in the series; specifically, suicide assessment and medication management were covered in a single session, which may have limited the opportunity to enhance knowledge or competence of medication management and prescription. It is also possible that knowledge gained from other sessions or clinical case presentations made individuals more aware of the totality of knowledge with respect to medication practices.

Any gains in knowledge from the single session on medication management may not have been enough to off-set a new perspective, producing lower scores. The possibility of this occurrence was identified in qualitative comments from respondents as many indicated how new knowledge increased their awareness of what they did not know. For example, a respondent stated that “[they] have become more aware of prevalence and how much [they] really don't know.” While perceived knowledge decreased slightly in these areas, the realization shared in the qualitative comments may serve as a catalyst for further learning.

The overall learning outcomes and improvements reported are likely to provide benefit to pediatric patients with behavioral health conditions in Idaho. The providers reported being better equipped to treat pediatric patients with behavioral health conditions through increased knowledge and competence with patient referrals, as well as improved overall competence in many clinical practice domains, including the use of evidence-based practice strategies and better education and care for patients and their families. Given the lack of providers in many locations across Idaho, these new resources will be valuable for optimizing patient/client care. As one respondent stated, the sessions “increased knowledge and connections within the medical community due to limited access to pediatric providers.” This respondent captured much of the ECHO Idaho series goals: create peer networking and learning opportunities for Idaho health care providers to optimize patient/client care.

### Limitations and future research

Despite this series resulting in many positive changes in health care providers' knowledge, competence, and confidence in different areas of pediatric behavioral health, the findings are not without limitations. The overall response rate for the presurvey was 10.8%, while the response rate for the postsurvey was 21.6%. Additionally, only seven total respondents completed both the pre- and postseries surveys, resulting in a small and likely less representative sample for this comparison. Future research should seek to obtain a higher response rate, as this would better identify representative patterns among all participants.

Additionally, data were collected immediately after the conclusion of the series, which may not have provided adequate time for some providers to implement learned information into practice (i.e., some respondents indicated that it was “too soon to tell” how the information changed their practice). Giving a longer period between the end of the series and the end of survey collection may be beneficial in future studies to allow implementation of new information into clinical practice.

Future research should also examine series duration to determine how session frequency, session duration, or series length influences attendance, peer learning, knowledge transfer, clinical practice change, and patient outcomes. It may be beneficial to determine if extending the series length, presenting a wider variety of topics and/or more in-depth discussion on the current topics, or utilizing a longer assessment longer period would impact program outcomes. An assessment of a longer duration series would also be more comparable to other Project ECHO series, and would allow for more direct comparison, and perhaps greater improvement in knowledge, competence, and confidence, especially given the promising results of this shorter series. Other participants mentioned that this information reinforced their clinical practice, but implied that they did not learn any new information. Future series could expand on the topics presented based on feedback from this subgroup of participants and researchers could assess impacts on knowledge.

## Conclusions

The ECHO Idaho Pediatric Behavioral Health 10-session series engaged 148 unique participants, with an average of 36 individuals per session. Most attendees (73%) were practicing Idaho health care providers with primary practices located in all seven Idaho PHDs. Overall, respondents were satisfied with individual sessions, believed attendance positively impacted their knowledge and clinical practice, and reported the knowledge and skills gained from the series would benefit more than half of their patients/clients.

Additionally, postseries respondents believed that the overall series provided new and relevant information; 95.8% of respondents believed their knowledge, competency, or practice changed because of their participation. Approximately 96.4% of postseries respondents believed that they are more effective at treating pediatric patients with behavioral health conditions because of their participation in the series. ECHO Idaho is a viable and valuable option to provide well-attended, well-received, and effective specialty training to Idaho providers to upscale knowledge and competence in providing care for pediatric patients with behavioral health conditions.

## Abbreviations Used

PCPs: primary care providers
Peds BH: Pediatric Behavioral Health
PHDs: public health districts
Project ECHO: Project Extension for Community Health Outcomes
SD: standard deviation
WWAMI: Washington, Wyoming, Alaska, Montana, Idaho

## References

[1] Leeb RT, Bitsko RH, Radhakrishnan L, et al. Mental health–related emergency department visits among children aged <18 years during the COVID-19 pandemic—United States, January 1–October 17, 2020. Morb Mortal Wkly Rep 2020;69(45):1675–1680; doi: 10.15585/mmwr.mm6945a3 PMC7660659 33180751

[2] de Miranda DM, da Silva Athanasio B, Sena Oliveira AC, et al. How is COVID-19 pandemic impacting mental health of children and adolescents?. Int J Disaster Risk Reduct 2020:101845; doi: 10.1016/j.ijdrr.2020.101845 32929399 PMC7481176

[3] Leff RA, Setzer E, Cicero MX, et al. Changes in pediatric emergency department visits for mental health during the COVID-19 pandemic: A cross-sectional study. Clin Child Psychol Psychiatry 2021;26(1):33–38; doi: 10.1177/1359104520972453 33183097

[4] Hill RM, Rufino K, Kurian S, et al. Suicide ideation and attempts in a pediatric emergency department before and during COVID-19. Pediatrics 2021;147(3):e2020029280; doi: 10.1542/peds.2020-029280 33328339

[5] Reinert M, Fritze D, Nguyen T. The State of Mental Health in America 2022. Mental Health America: Alexandria VA; 2021; 45 p.

[6] Sareen J, Jagdeo A, Cox BJ, et al. Perceived barriers to mental health service utilization in the United States, Ontario, and the Netherlands. Psychiatr Serv 2007;58(3):357–364; doi: 10.1176/ps.2007.58.3.357 17325109

[7] Ghandour RM, Sherman LJ, Vladutiu CJ, et al. Prevalence and treatment of depression, anxiety, and conduct problems in US children. J Pediatr 2019;206:256–267; doi: 10.1016/j.jpeds.2018.09.021 30322701 PMC6673640

[8] Jimenez DE, Bartels SJ, Cardenas V, et al. Cultural beliefs and mental health treatment preferences of ethnically diverse older adult consumers in primary care. Am J Geriatr Psychiatry 2012;20(6):533–542; doi: 10.1097/JGP.0b013e318227f876 21992942 PMC3258470

[9] Holder SM, Peterson ER, Stephens R, et al. Stigma in mental health at the macro and micro levels: Implications for mental health consumers and professionals. Commun Mental Health J 2019;55(3):369–374; doi: 10.1007/s10597-018-0308-y 30069706

[10] Horwitz SM, Storfer-Isser A, Kerker BD, et al. Barriers to the identification and management of psychosocial problems: Changes from 2004 to 2013. Acad Pediatr 2015;15(6):613–620; doi: 10.1016/j.acap.2015.08.006 26409303 PMC4639452

[11] Whalen L, Moehrle C. Community Health Assessment. Panhandle Health District and Idaho North Central District: Hayden, ID; 2013; 50 p.

[12] Children's Mental Health. Centers for Disease Control and Prevention: Atlanta, GA; 2021. Behavioral Health Services in Idaho; 2021; [about 3 screens]. Available from: https://www.cdc.gov/childrensmentalhealth/stateprofiles-providers/idaho/index.html [Last accessed: February 2, 2022].

[13] Substance Abuse and Mental Health Services Administration. Behavioral health barometer, Idaho, Volume 4: Indicators as measured through the 2015 National Survey on Drug Use and Health, the National Survey of Substance Abuse and Treatment Services, and the Uniform Reporting System. Substance Abuse and Mental Health Services Administration: Rockville, MD; 2017; 24 p. Report No.: SMA-17-Baro-16-States-ID. 33734653

[14] Vannoy SD, Tai-Seale M, Duberstein P, et al. Now what should I do? Primary care physicians' responses to older adults expressing thoughts of suicide. J Gen Internal Med 2011;26(9):1005–1011; doi: 10.1007/s11606-011-1726-5 21541796 PMC3157512

[15] UNM health sciences: Project ECHO. University of New Mexico: Albuquerque, NM; c2022. About Project ECHO; 2022; [about 2 screens]. Available from: https://hsc.unm.edu/echo/about-us [Last accessed: February 2, 2022].

[16] University of Idaho. University of Idaho: Moscow, ID; c2022. ECHO Idaho; 2022; [about 2 screens]. Available from: https://www.uidaho.edu/academics/wwami/echo [Last accessed: February 2, 2022].

[17] Baker RT, Casanova MP, Whitlock JN, et al. Expanding access to health care: Evaluating project Extension for Community Health Care Outcomes (ECHO) Idaho's tele-education behavioral health program. J Rural Mental Health 2020;44(4):205–216; doi: 10.1037/rmh0000157

[18] Casanova MP, Nelson MC, Blades KC, et al. Evaluation of an opioid and addiction treatment tele-education program for healthcare providers in a rural and frontier state. J Opioid Manage 2022;18(4):297–308; doi: 10.5055/jom.2022.0725 36052928

[19] Thomas DR. A general inductive approach for analyzing qualitative evaluation data. Am J Eval 2006;27(2):237–246; doi: 10.1177/1098214005283748

[20] Sockalingam S, Arena A, Serhal E, et al. Building provincial mental health capacity in primary care: An evaluation of a project ECHO mental health program. Acad Psychiatry 2018;42(4):451–457; doi: 10.1007/s40596-017-0735-z 28593537

[21] Lalloo C, Osei-Twum JA, Rapoport A, et al. Pediatric Project ECHO^®^: A virtual community of practice to improve palliative care knowledge and self-efficacy among interprofessional health care providers. J Palliat Med 2021;24(7):1036–1044; doi: 10.1089/jpm.2020.0496 33326309 PMC8215401

[22] Hostutler CA, Valleru J, Maciejewski HM, et al. Improving pediatrician's behavioral health competencies through the project ECHO teleconsultation model. Clin Pediatr 2020;59(12):1049–1057; doi: 10.1177/0009922820927018 32506939

[23] Curran VR, Fleet L, Kirby F. Factors influencing rural health care professionals' access to continuing professional education. Aust J Rural Health 2006;14(2):51–55; doi: 10.1111/j.1440-1584.2006.00763.x 16512789

[24] Hancock C, Steinbach A, Nesbitt TS, et al. Why doctors choose small towns: A developmental model of rural physician recruitment and retention. Soc Sci Med 2009;69(9):1368–1376; doi: 10.1016/j.socscimed.2009.08.002 19747755

[25] Nhung LH, Dien TM, Lan NP, et al. Use of Project ECHO Telementoring model in continuing medical education for pediatricians in Vietnam: Preliminary results. Health Serv Insights 2021;14:11786329211036855; doi: 10.1177/11786329211036855 34408433 PMC8366124

[26] Kim MK, Arsenault C, Atuyambe LM, et al. Determinants of healthcare providers' confidence in their clinical skills to deliver quality obstetric and newborn care in Uganda and Zambia. BMC Health Serv Res 2020;20(1):539; doi: 10.1186/s12913-020-05410-3 32539737 PMC7296707

[27] Vandergrift JL, Gray BM. Physician clinical knowledge, practice infrastructure, and quality of care. Am J Managed Care 2019;25(10):497–503. PMID: 31622065

